# Potential of Commercial Biorational and Conventional Pesticides to Manage the Ruellia Erinose Mite in Ornamental Landscapes

**DOI:** 10.3390/insects16080801

**Published:** 2025-08-02

**Authors:** Marcello De Giosa, Adam G. Dale, Xingbo Wu, Alexandra M. Revynthi

**Affiliations:** 1 Tropical Research and Education Center, Entomology and Nematology Department, Institute of Food and Agricultural Sciences, University of Florida, Homestead, FL 33031, USA; 2 Entomology and Nematology Department, Institute of Food and Agricultural Sciences, University of Florida, Gainesville, FL 32611, USA; agdale@ufl.edu; 3 Tropical Research and Education Center, Environmental Horticulture Department, Institute of Food and Agricultural Sciences, University of Florida, Homestead, FL 33031, USA

**Keywords:** integrated pest management, curative application, prophylactic application, chemical control, Eriophyoidea

## Abstract

*Acalitus simplex* is an eriophyoid mite pest of the ornamental plant *Ruellia simplex*. *Acalitus simplex* compromises the esthetics of *R. simplex* by inducing erinea formation. Management practices for *A. simplex* are currently lacking. This study assessed the potential of commercial biorational (citric acid, potassium salt of fatty acids, garlic, thyme, and mineral oil) and conventional (abamectin, fenpyroximate, bifenthrin, spiromesifen) pesticides under laboratory conditions, using two types of spray applications: (A) curative, after erinea formation, and (B) prophylactic, before erinea formation. In the curative application, abamectin, garlic oil, and mineral oil were most effective; in the prophylactic application, abamectin and mineral oil showed the highest efficacies. Abamectin and mineral oil were further tested under greenhouse conditions. Both treatments effectively controlled *A. simplex* by preventing erinea formation over a four-week post-application period, regardless of the application type. At the end of the experiment, mites were extracted from *R. simplex* plants. In the curative application, significantly fewer mites were extracted from abamectin and mineral oil treatments than in the control. In the prophylactic application, mites were absent in abamectin and mineral oil treatments but present in the control. Abamectin and mineral oil can be used to manage *A. simplex* in landscapes.

## Simple Summary

The Ruellia erinose mite, *Acalitus simplex*, is a pest that damages the ornamental plant *Ruellia simplex* by inducing the formation of galls, known as erinea. Currently, management tools to mitigate this pest are underdeveloped. This study aimed to test various commercial pesticides and spray application methods to determine which could prevent and reduce mite infestations on *Ruellia simplex*. Abamectin and mineral oil were found to be highly effective in both preventing new infestations from the Ruellia erinose mite and reducing existing ones. These findings provide practical solutions for preserving the esthetic value of *R. simplex* in both commercial and residential landscapes while minimizing risks to humans, pets, and the environment.

## 1. Introduction

Ornamental landscapes, hereafter landscapes, encompass a variety of outdoor spaces, from urban areas such as golf courses, city parks, condominiums, commercial and tourist complexes, as well as residential properties [1]. Vegetation in these spaces provides multiple ecosystem services and functions, including social, esthetic, hydrological, ecological, and economic benefits [2,3]. Among the many plants that shape ornamental landscapes, species in the genus *Ruellia* L. are commonly planted for their diverse flower sizes and colors, as well as their resilience to various environmental stresses [4]. *Ruellia simplex* Wright (Lamiales: Acanthaceae), commonly known as Mexican petunia, or Britton’s wild petunia, is the most widely ornamental *Ruellia* species, cultivated and planted in residential and commercial landscapes in the southern United States [4]. *Ruellia simplex* is a perennial shrub that was previously classified as an invasive plant species in several regions, including Florida. Nowadays, *R. simplex* is no longer listed as invasive and is considered a naturalized species in Florida due to the establishment of wild populations across 35 counties and in 22 natural areas [4,5,6,7]. Despite its previous invasive status, the Florida Nursery, Growers and Landscape Association (FNGLA) recognizes *R. simplex* as a valuable ornamental plant, with estimated annual sales of approximately $12 million in the ornamental plant industry [4,7].

*Ruellia simplex* is susceptible to infestation by the Ruellia erinose mite, *Acalitus simplex* Flechtmann and Etienne (Acari: Eriophyidae), which can severely reduce its esthetic quality, appeal to consumers, and ecosystem service contribution to landscapes [8,9,10,11,12]. *Acalitus simplex* is a gall-making eriophyid mite that exclusively feeds on plant species within the genus *Ruellia* [13]. Although *A. simplex* has been observed on other species, including *Ruellia blechum* L., *Ruellia caroliniensis* (Small) Long, *Ruellia squarrosa* (Oerst.) Hemsl., and *Ruellia tuberosa* L., it has been mainly reported on *R. simplex* [10,11,12,13]. The feeding activity of *A. simplex* induces the formation of open galls, known as erinea [14], leading to distortion and twisting of the affected plant tissue [15,16]. The erinea, which resemble hair patches [17], can form on various *R. simplex* tissues, including petioles, leaves, pedicels, sepals, flower buds, and flowers, partially or entirely covering these structures [13]. On these plant tissue, erinea develop on both adaxial and abaxial surfaces, with no apparent preference for either side [13]. The erinea progress through three to four distinct stages, each defined by a characteristic color: hyaline (stage 1), white (stage 2), purple (stage 3), and beige (stage 4) [13]. The population density of *A. simplex* varies across the erinea stages, peaking in the white and purple stages and declining in the hyaline and beige stages [13]. The formation of the erinea provides several advantages to eriophyoid mites [17]. Erinea serve as a nutrient source [18,19] and as shelters that allow mites to conceal themselves and avoid predation by natural enemies [20,21,22]. In addition, erinea can interfere with chemical control applications because the hydrophobic properties of the erineum hairs cause pesticide droplets to run off rather than adhere and penetrate the surface, preventing direct pesticide exposure to the mites [23,24].

To manage mites and other arthropod infestations sustainably, integrated pest management (IPM) strategies are commonly employed in the landscape [25]. These strategies involve pest identification, monitoring, decision-making, intervention, and evaluation [26]. Due to knowledge gaps regarding the biology and ecology of most known species, intervention against eriophyoid mites primarily relies on chemical control [27,28,29,30]. Because eriophyoid mites are concealed within erinea [24], curative treatments applied to existing erinea have limited efficacy, as the erinea hinder pesticide penetration, preventing contact with the mites or their eggs and allowing populations to persist after treatment [31,32]. Therefore, eriophyoid mites are more effectively controlled with preventive pesticide applications before the erinea are formed. Despite their low efficacy, curative pesticide applications are often necessary because eriophyoid mites, measuring only 86 to 500 µm in length [33], are usually detected only after fully developed galls have formed on the plants [34]. Such applications may still reduce the spread and prevent the mites from infesting new tissues on the treated plant. Effective curative pesticide applications are often limited to periods when mites emerge from the erinea, typically during their dispersal to infest new plant tissues [27]. Because dispersal usually occurs over several days or weeks [35], pesticides with extended residual activity are most effective in preventing reinfestation [28,32].

Currently, information on the chemical control of *A. simplex* is underdeveloped, thereby limiting the number of tools for management. Considering the concealed lifestyle of *A. simplex*, we tested two hypotheses: (i) preventive pesticide applications are more effective than curative treatments in inhibiting *A. simplex* infestations and establishment on the plant, and (ii) curative applications can reduce *A. simplex* population within the erinea and prevent colonization of new plant tissue due to the residual activity of the pesticides. To test these hypotheses, this study determined the efficacy of several formulated biorational and conventional pesticides (Table 1), representing distinct Insecticide Resistance Action Committee (IRAC) chemical classes [36], applied as prophylactic or curative sprays, to mitigate *A. simplex* on *R. simplex*. The findings of this study provide insights into the most effective formulated pesticides and the most appropriate application to incorporate into IPM strategies for controlling *A. simplex* infestations on *R. simplex* in the landscape.

## 2. Materials and Methods

### 2.1. Ruellia Simplex Stock Population

*Ruellia simplex* plants were grown from seeds (Southern Start Blue, Salem, OR, USA) in 1 L seedling trays filled with soil (ProMix BX Mycorhizae, Denver, CO, USA) and maintained for germination in a climate-controlled room at 25 ± 2 °C, 50% RH, and 12:12 h (L:D). Seedlings were watered three times per week to maintain adequate moisture. Once the *R. simplex* reached approximately 6 cm in stem length, they were transplanted into 3.7 L pots filled with soil and placed in insect-proof mesh cages (47.5 × 47.5 × 93.0 cm, mesh size 140 µm, BugDorm–BD4M4590, Taichung, Taiwan) to prevent pest infestations. *Ruellia simplex* plants were fertilized twice a month with 24–8–16 (N–P–K) (Miracle–Gro, The Scotts Company, Marysville, OH, USA) and 138 mg/L chelated EDDHA iron (Sequestrene, Syngenta, Wilmington, DE, USA).

### 2.2. Acalitus Simplex Stock Population

An *A. simplex* colony was established using leaves and stems with white erinea (stage 2) from infested *R. simplex* plants, because this stage contains the highest number of mites [13]. Infested *R. simplex* samples were collected from outdoor landscape plantings at the University of Florida’s Tropical Research and Education Center (TREC) in Homestead, FL (25°30′30″ N, 80°29′55″ W). Leaves and stems were placed on black plastic tiles (15 × 8 cm) supported by a cellulose sponge cube (19.05 × 10.92 × 5 cm, 3M, St. Paul, MN, USA) inside plastic boxes (22.5 × 15 × 5 cm, The Container Store, Orlando, FL, USA) partially submerged in water. After 24 h, *A. simplex* individuals began to emerge from the erinea, facilitating their collection [30]. *Acalitus simplex* individuals were collected using a camel-hair brush (Blick master synthetic round 3.0) under a stereomicroscope (MZ6, Leica, Wetzlar, Germany) and transferred to *R. simplex* plants, approximately 6 cm tall, sourced from the stock population. *Acalitus simplex* stock population was maintained in a growth chamber (Conviron model No. PGW40, Winnipeg, MB, Canada) under controlled conditions of 25 ± 2 °C, 50 ± 5% RH and photoperiod 12:12 h (L:D). *Ruellia simplex* plants were watered three times per week and fertilized twice a month, as described previously.

### 2.3. Infestations of Ruellia Simplex Plants for Laboratory and Greenhouse Experiments

Leaves with white erinea were sampled from the *A. simplex* stock population to establish a cohort of infested plants. Thirty *A. simplex* individuals were collected to initiate a substantial infestation [13]. These mites were transferred to the new shoots of each *R. simplex* plant sourced from the stock population, using the methodology described in Section 2.2. The newly infested plants were maintained in a growth chamber under controlled conditions, following the procedures described above. These plants were kept in the growth chamber until the erinea turned white, which occurred in approximately 15 days [13], before being used in laboratory or greenhouse experiments. We targeted the white erinea (stage 2) because hyaline erinea (stage 1) are often undetectable to the naked eye due to the transparency of their hairs and resemblance to plants natural trichomes [13].

### 2.4. Pesticide Testing in the Laboratory

Under laboratory conditions, ten treatments were evaluated, including four conventional and five biorational pesticides. Water was included as control. Pesticides were selected based on the following criteria: (i) suitability for applications on ornamental plants in the U.S., (ii) target pest listed on the label (gall–makers, eriophyid species, and mites generally), (iii) approved application to landscape and residential sites where *R. simplex* plants may be encountered in the U.S., and (iv) registration of the pesticides in Florida’s National Pesticide Information Retrieval System database [37]. Pesticide solutions were prepared at the maximum label rate recommended for controlling mites or eriophyoids (if available) in 50 mL of water (Table 1). For both curative and prophylactic applications, 0.4 mL of each pesticide solution (mean of applied solution: 0.42 mg/cm^2^) was applied using a Potter Spray Tower (Burkard Manufacturing Co., Ltd., Rickmansworth, UK) at 5 PSI pressure. The tower was cleaned between treatments with 1000 µL of pure acetone (Fisher chemical, cat. no A18–4, Pittsburg, PA, USA), 70% ethanol (Fisher bioreagents, cat. no bp2818–4, Pittsburg, PA, USA), and distilled water, followed by drying with paper towels.

#### 2.4.1. Curative Spray Application Tests

Curative spray applications were performed on 0.5 cm diameter leaf disks, covered with white erinea, sourced from infested *R. simplex* plants using a cork borer (Fisherbrand, Pittsburg, PA, USA). Ten leaf disks (replicates), each collected from a different plant, were used for each treatment, and the entire experiment was repeated three times (blocks). Each leaf disk was individually placed in a Petri dish (5.5 cm diameter, BD Falcon, Mexico City, Mexico) beneath the Potter Spray Tower. After application, the leaf disk was transferred to a black plastic tile (6 × 6 cm) and double-sided tape (1.25 cm wide; 3M, Miami, FL, USA) was applied along the edges of the tile to contain *A. simplex* from escaping. The black plastic tiles were then placed in trays under controlled conditions at 25 ± 2 °C, 50 ± 5% RH and photoperiod 12:12 h (L:D). Due to the difficulty in directly observing *A. simplex* motile stages or eggs within the erineum, pesticide efficacy was assessed by recording mite emergence from the erineum at 24, 48, and 72 h post-application. This evaluation timeframe was chosen based on previous findings indicating that *A. simplex* emergence is highest on the first day, followed by reduced numbers on the second and third day [13]. Efficacious pesticides were expected to result in fewer or no emerging *A. simplex*.

#### 2.4.2. Prophylactic Spray Application Tests

Prophylactic spray applications were also performed on 0.5 cm leaf disks, but those without erinea that were sourced from the *R. simplex* stock population. An equal number of replicates and blocks were conducted as in the previous experiments. Each leaf disk was individually placed in an RNase-Free tube (Eppendorf, Thermo Fisher Scientific, Woodward St., Austin, Texas USA) containing 1.5 mL of 1% agar (A360–500, Fisher Scientific, Madrid, Spain). The lid was modified with an opening (0.4 mm in diameter), to which a fine mite-proof mesh (mesh diameter 36 µm, Woven Wire Mesh, Inoxia, Cranleigh, UK) was attached to ensure ventilation throughout the experiment. Tubes were positioned beneath the Potter Spray Tower to ensure complete pesticide coverage of both the leaf surface and the internal tube walls, where *A. simplex* could walk. After application, tubes were left to dry on a tube rack under controlled conditions at 25 ± 2 °C, 50 ± 5% RH, and a photoperiod of 12:12 h (L:D). At 24 h post-application, 30 *A. simplex* were transferred to each leaf disk inside the tube. The tube rack was then returned to the same controlled conditions. Pesticide efficacy was assessed by monitoring *A. simplex* mortality at 24, 48, and 72 h after mite release on the leaf disk. Efficacious pesticides were expected to result in higher *A. simplex* mortality.

### 2.5. Greenhouse Evaluation of Top Lab-Selected Pesticides

The most efficacious conventional (abamectin) and biorational (mineral oil) formulated pesticides identified in the laboratory experiments (results Section 3.1), were further evaluated under greenhouse conditions (25 ± 5 °C; 70 ± 20% RH) from late September 2024 to early January 2025, with water serving as the control. Pesticide solutions were prepared at the maximum recommended label rates (Table 1) in 1500 mL of water and applied until runoff using 1.75 L HDX™ handheld sprayers (Root–Lowell Manufacturing Co., Lowell, MI, USA). For both curative and prophylactic applications, *R. simplex* plants approximately 6 cm tall, with white erinea and plants without erinea were placed in mite-proof mesh cages (W 32.5 × D 32.5 × H 77.0 cm, mesh diameter 160 µm; BD4E3074 BugDorm, Taiwan) one week before pesticide application to ensure plant acclimation to greenhouse conditions. To minimize potential microclimate variation due to uneven exposure to greenhouse features (e.g., ventilation, sunlight), cages containing plants exhibiting erinea and cages with plants without erinea were arranged in an alternating pattern and randomized within each block. This randomized complete block design ensured that all treatments were equally distributed across microclimate gradients within the greenhouse, thereby minimizing positional effects on plant growth, *A. simplex* infestation, and pesticide efficacy. An automatic drip irrigation system provided water for five minutes weekly, with fertilizer twice a month, as described above. Each treatment included ten *R. simplex* plants (replicates) per application type, with the experiment repeated three times (blocks). In the curative spray, one day before pesticide application, leaves, stems, buds, and flowers with white erinea were colored marked with a permanent marker (Sharpie, fine point, Atlanta, GA, USA) to avoid recounting them during subsequent evaluations. In the prophylactic spray, 24 h post-application, 30 *A. simplex* individuals were transferred to each *R. simplex* plant. The efficacy of the treatments and applications was assessed by recording the number of stems, leaves, buds, and flowers that developed erinea weekly, for four weeks after pesticide application (curative) or mite release (prophylactic). Newly formed plant tissues that developed erinea were colored with the permanent marker to avoid recounting them in the following weekly evaluation. The weekly interval was chosen based on previous findings indicating that hyaline erinea develop within approximately ten days of *A. simplex* establishment [13]. The four-week duration was selected because white erinea present on the plants gradually turn beige within approximately 40 days, a stage associated with a significantly reduced mite population [13]. Efficacious pesticides and applications were expected to result in a lower number of plant tissue developing erinea compared to the control.

To further assess the efficacy of the evaluated pesticides and applications in the greenhouse, destructive samplings of entire *R. simplex* plants were conducted at the end of each experiment. Plant samples, including stems, leaves, buds, and flowers, were washed following the methodology described by Monfreda et al. [38] to extract and quantify *A. simplex* individuals. Mites were counted in a grid-marked Petri dish (13.5 cm in diameter, Fisher), which had 32 full squares (2 × 2 cm) and 20 partial squares (2 × 1 cm) along the border. Counts were performed under a stereoscope by randomly selecting three full squares per plant sample.

### 2.6. Statistical Analysis

Statistical analyses were performed in RStudio version 2024.12.0 [39]. Each application, either curative or prophylactic, was analyzed independently within its respective experimental settings (laboratory, greenhouse and extraction) due to differences in mite quantification and infestation density at the time of treatment. In the prophylactic spray application, the number of *A. simplex* transferred was set at 30 individuals, whereas in the curative spray application, the initial infestation was standardized at 30 *A. simplex* individuals per plant; however, population growth over the four-week period preceding pesticide application was not quantified.

#### 2.6.1. Pesticide Testing in the Laboratory

The curative and prophylactic spray applications conducted under laboratory conditions were analyzed with a generalized linear mixed-effects model (GLMM), with negative binomial error distribution using the glmmTMB package [40]. In the curative application, the cumulative number of live *A. simplex* emerging from the erinea was the response variable, whereas in the prophylactic application, the response variable was the cumulative number of dead *A. simplex*. In both analyses, treatments, time since treatment application, and their interaction were the independent variables, with block as a random intercept effect. Post hoc tests were performed using the estimated marginal means (EMMs) with Tukey’s HSD adjustment, implemented in the emmeans package [41]. Graphs were generated using the ggplot2 package [42].

#### 2.6.2. Greenhouse Evaluation of Top Lab-Selected Pesticides

Models comparing *A. simplex* counts for both the curative and prophylactic spray application experiments under greenhouse conditions did not meet the assumptions of a parametric model and were therefore analyzed using the Kruskal–Wallis nonparametric test (α = 0.05). Pairwise comparisons were performed using the Wilcoxon test [43]. The cumulative number of tissues developing erinea per plant was the response variable, and the interaction between treatments and evaluation time were the explanatory variables. Graphs were generated using the ggplot2 package.

Similarly, data from the extraction assays were analyzed using the Kruskal–Wallis nonparametric test. The number of extracted *A. simplex* individuals was the response variable, and treatment was the explanatory variable. Post hoc tests were performed using the Wilcoxon test.

## 3. Results

### 3.1. Pesticide Testing in the Laboratory

#### 3.1.1. Curative Spray Application Tests

**Figure 1:**
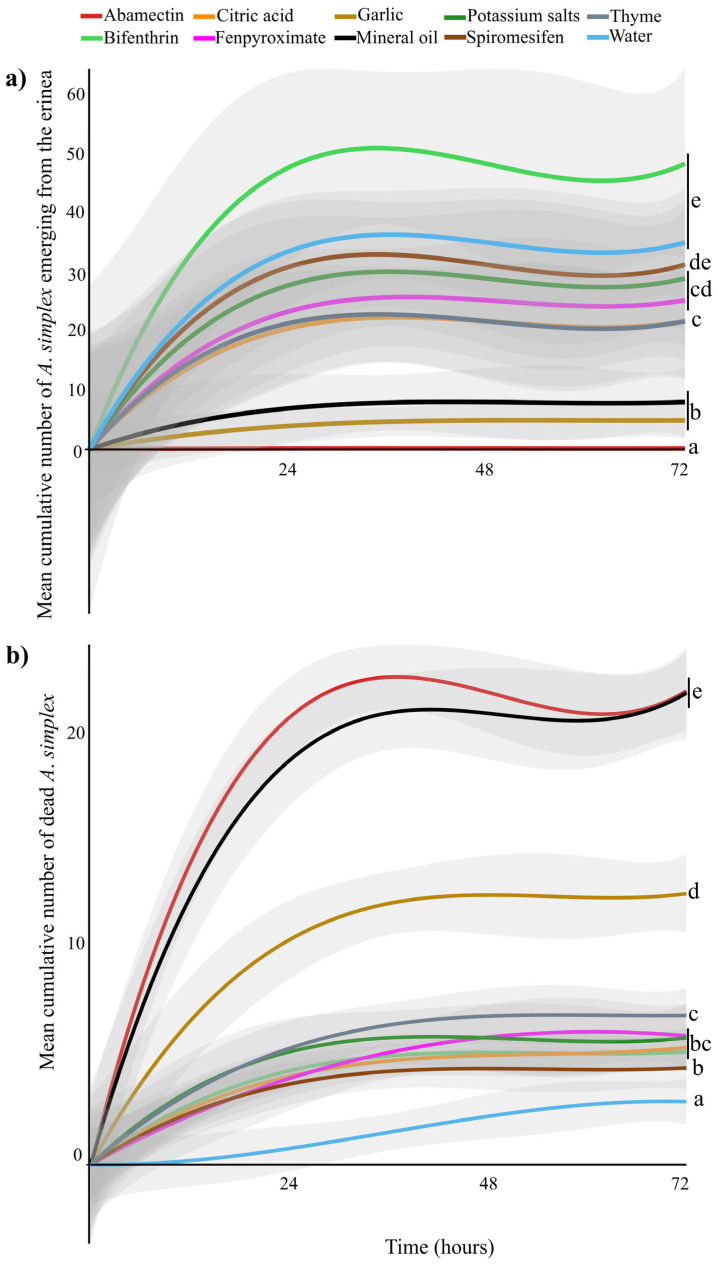
(**a**) Mean cumulative number of *Acalitus simplex* emerging from white erinea over time (hours) after curative applications; (**b**) Mean cumulative number of dead *A. simplex* over time after prophylactic applications. Statistically different treatments are separated with lowercase letters (N = 30 for each treatment, GLMM, *p* < 0.05). Gray shaded areas represent the predicted standard error, generated by R program.

Direct exposure of the erinea to pesticides resulted in a significant reduction in emerging *A. simplex* (GLMM: *χ*^2^ = 319.98 df = 9, *p* < 0.001). Time since pesticide exposure did not affect the number of emerging mites (GLMM: *χ*^2^ = 0.80 df = 2, *p* = 0.66), nor did the interaction between treatment and time (GLMM: *χ*^2^ = 0.57 df = 10, *p* = 1). Pairwise comparisons of treatment means indicated that abamectin resulted in the fewest *A. simplex* emerging from the white erinea 0.16 ± 0.04 (mean ± SE) compared to all other treatments (*p* < 0.001). Garlic (4.61 ± 0.93) and mineral oil (7.67 ± 1.76) did not differ significantly from each other (*p* = 1), but both differed significantly from thyme oil (21.61 ± 3.01), citric acid (21.32 ± 2.88), fenpyroximate (24.65 ± 3.09), potassium salts of fatty acids (28.60 ± 3.45), and spiromesifen (31.18 ± 3.34) (*p* < 0.001). The highest numbers of emerging *A. simplex* from the erinea were observed in the water (34.57 ± 2.79) and bifenthrin (48.20 ± 4.85), which were statistically similar to each other (*p* = 0.94) (Figure 1a, Table 2).

#### 3.1.2. Prophylactic Spray Application Tests

Prophylactic application of pesticides also resulted in significant mortality of *A. simplex* (GLMM: *χ*^2^ = 1783.29 df = 9, *p* < 0.001). Furthermore, mortality varied with time after application (GLMM: *χ*^2^ = 24.63 df = 2, *p* < 0.001), with higher mortality observed within 24 h post-application compared to 48 h (*p* < 0.001) and 72 h (*p* < 0.001). No significant difference in mortality was detected between 48 and 72 h (*p* = 0.56). Pesticide treatments and time did not have a combined effect on mite mortality (GLMM: *χ*^2^ = 20.723 df = 18, *p* = 0.29). Abamectin (22.08 ± 0.58) (mean± SE) and mineral oil (21.04 ± 0.67) caused the highest mite mortality, and their effects did not differ significantly from each other (*p* = 0.96). Garlic oil (12.14 ± 0.57) caused lower mortality than abamectin and mineral oil (*p* < 0.0001). Garlic oil, however, caused significantly higher mortality than thyme oil (6.56 ± 0.40), potassium salts of fatty acids (5.81 ± 0.48), bifenthrin (5.05 ± 0.27), fenpyroximate (5.43 ± 0.47), citric acid (4.98 ± 0.30), spiromesifen (4.33 ± 0.30), and water (2.02 ± 0.34) (*p* < 0.0001) (Figure 1b, Table 2).

### 3.2. Greenhouse Evaluation of Top Lab-Selected Pesticides

**Figure 2:**
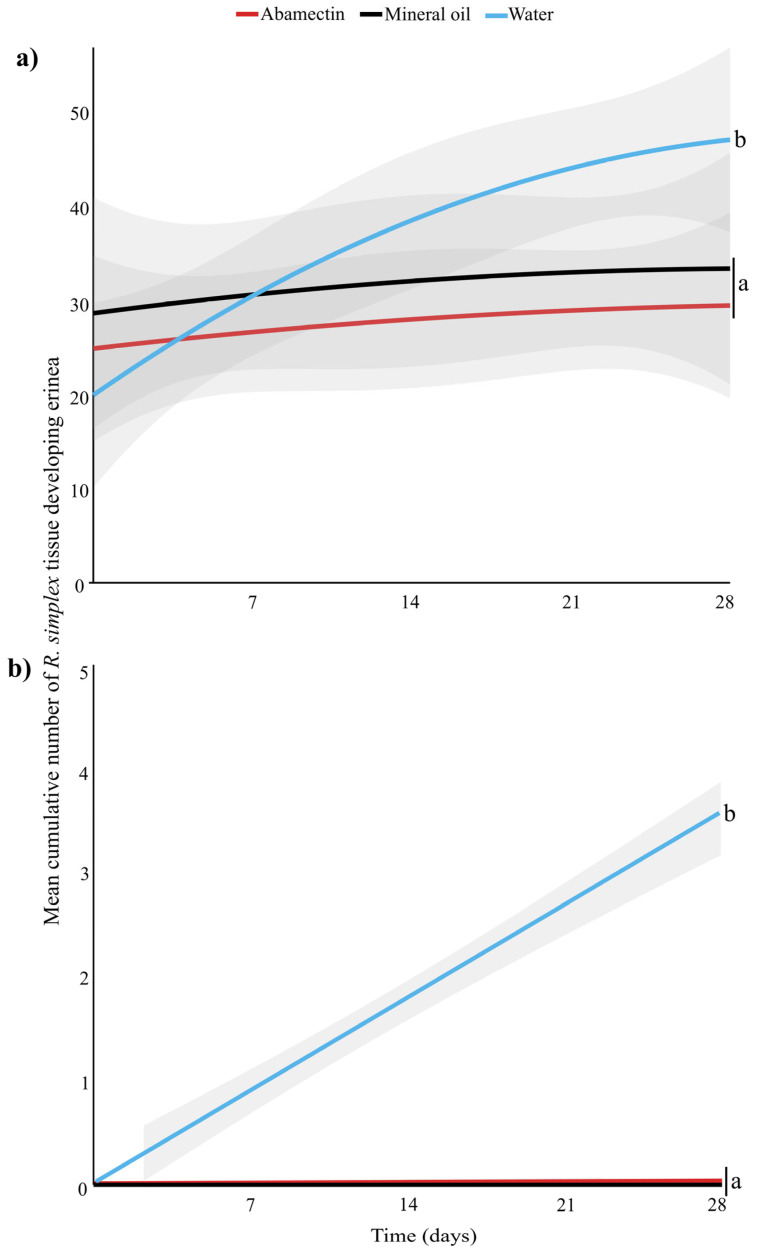
Mean cumulative number of *Ruellia simplex* tissue (stems, leaves, buds and flowers) developing erinea over time (days) after (**a**) curative application and (**b**) prophylactic treatment application. Statistically different treatments are separated with lowercase letters (N = 30 for each treatment, Kruskal–Wallis, *p* < 0.05). Gray shaded areas represent the predicted standard error, generated by R program.

The effect of pesticide treatment on the number of newly formed erinea varied over time in both curative (Kruskal–Wallis: *χ*^2^ = 52.65, df = 14, *p* < 0.001) (Figure 2a) and prophylactic applications (Kruskal–Wallis: *χ*^2^ = 408.4, df = 14, *p* < 0.001) (Figure 2b). In the curative application of both abamectin and mineral oil, new erinea began forming on new plant tissue approximately two weeks post-application, after which the number of plant tissues forming new erinea ceased. In the prophylactic application, both abamectin and mineral oil protected plant tissue, resulting in no erinea formation throughout the four-week period.

**Figure 3:**
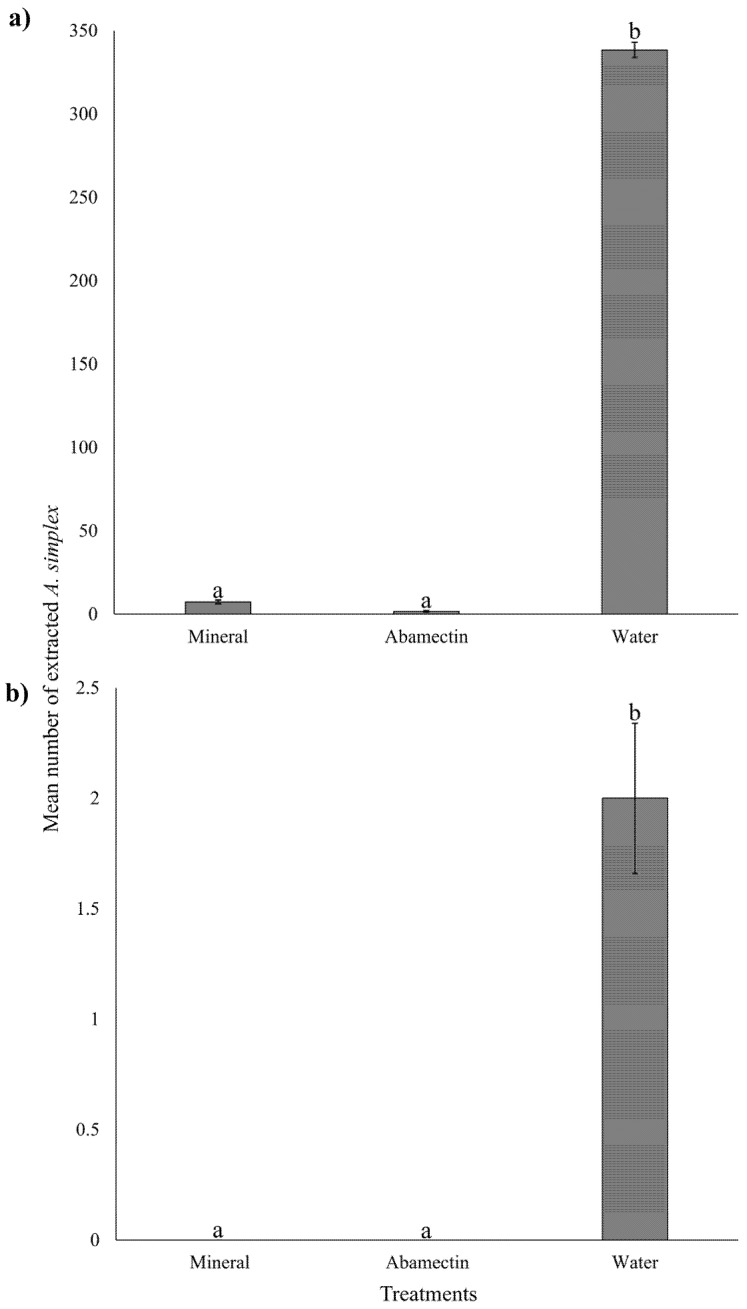
(**a**) Mean number of *Acalitus simplex* extracted from sprayed *Ruellia simplex* with curative and (**b**) prophylactic applications. Statistically different treatments are separated with lowercase letters (Kruskal–Wallis, *p* < 0.05). Error bars indicate the standard error of the mean, with N = 30 for each treatment.

In curative application, the mean number of extracted *A. simplex* from plants treated with abamectin (1.56 ± 0.42) (mean ± SE) and mineral oil (7.33 ± 1.10) was significantly lower than plants treated with water (338.4 ± 4.65) (Kruskal–Wallis: *χ*^2^ = 59.35, df = 2, *p* < 0.001) (Figure 3a). In the prophylactic application, the number of extracted mites also varied with treatment (Kruskal–Wallis: *χ*^2^ = 22.15, df = 2, *p* < 0.001). No mites were extracted from the abamectin and mineral oil treatments, whereas a low number of mites was extracted from the water-treated plants (2 ± 0.34) (Figure 3b).

## 4. Discussion and Conclusions

*Acalitus simplex* mites are a major pest of the ornamental plant *R*. *simplex*, commonly planted in landscapes. *Acalitus simplex* mite infestations compromise the esthetic value of *R. simplex* plants by inducing the formation of erinea on stems, leaves, buds, and flowers [13]. Currently, no established chemical control practices are available for managing this mite pest in the landscape. To address this knowledge gap, we evaluated the efficacy of five biorational and four conventional formulated pesticides against *A. simplex* using two spray strategies: a curative and a prophylactic application. Our results from both laboratory and greenhouse experiments fully corroborate the tested hypotheses by demonstrating that prophylactic applications of specific pesticides are more effective than curative treatments, as reflected by consistently lower mean mite counts and narrower confidence intervals (Table 2, Figure 1). Nonetheless, curative treatments are also effective in reducing mite populations within already developed erinea and in preventing new infestations, thereby allowing partial restoration of their ornamental value. Among the nine evaluated pesticides, one conventional (abamectin) and two biorational (mineral oil and garlic oil) products are highly effective in controlling *A. simplex* through both curative and prophylactic applications under laboratory conditions. Abamectin and mineral oil were selected for further evaluation under greenhouse conditions, due to their consistently high efficacy when administered curatively and preventively. Curative applications of abamectin and mineral oil effectively reduced the development of erinea on plant tissues (Figure 2a) by decreasing the population of *A. simplex* within the erinea (Figure 3a). Prophylactic applications prevented new infestations (Figure 2b) by suppressing *A. simplex* individuals before colonization occurred (Figure 3b). Overall, our results provide promising evidence that chemical tools can play a valuable role in *A. simplex* mite IPM programs to preserve the esthetic and economic value of *R. simplex* plants in ornamental systems.

Abamectin is effective against motile mite stages, such as nymphs and adults, and acts through direct contact and ingestion, affecting the nervous system and causing paralysis and death in exposed mites [36]. Although not systemic, abamectin exhibits translaminar properties [44], allowing the active ingredient to penetrate the epidermis of buds, galls (i.e., erinea), and other plant tissues, providing relatively long residual activity [45]. This residual activity is particularly effective against eriophyoids, that have short stylets (approximately 20 µm or less) and thus feed on epidermal cells where the active ingredient accumulates, ultimately leading to their control [23,46]. In our study, abamectin reduced *A. simplex* populations within the erinea and exhibited residual activity lasting four weeks, preventing the formation of new erinea on developing plant tissue. Mineral oil is effective against both eggs and motile stages, primarily by suffocating pests through interference with the respiratory system [47]. Additionally, it disrupts cuticular waxes, softens the exoskeleton, and causes dehydration [48]. Furthermore, mineral oil acts as a feeding and oviposition deterrent [49]. The residual film formed on the plant surface may also prevent eriophyoid mites from attaching and feeding, similar to its effects on hemipteran nymphs [49]. Consistent with these mechanisms, mineral oil prevented new *A. simplex* infestations on developing plant tissue throughout the four-week period. Abamectin and mineral oil have been documented as effective against other gall-forming eriophyoid mites, including *Aceria litchii* (Keifer) and *Eriophyes dioscoridis* Soliman and Abou–Awad [50,51], as well as other phytophagous mites like *Tetranychus urticae* Koch and *Raoiella indica* Hirst [52,53]. Our findings expand this evidence by demonstrating their efficacy against *A. simplex*, with curative applications reducing mite emergence and associated erinea formation, and prophylactic applications achieving complete suppression of infestations. Moreover, both abamectin and mineral oil are labeled for use on ornamentals against a broad range of insect pests, including aphids, leafminers, scales, thrips, weevils and whiteflies [54,55,56,57]. This provides an additional advantage, as *R. simplex* in landscapes is often exposed to pests beyond *A. simplex*, such as flat mites (*Brevipalpus* spp.) [58] and scale insects, like *Ceroplastes cirripediformis* Comstock, *Pulvinaria urbicola* Cockerell, and *Ferrisia virgata* (Cockerell) [59] that can also compromise the plant’s esthetic value. The novel findings of this study support the integration of chemical control strategies into *A. simplex* management programs in ornamental landscapes. Because ornamental landscapes are often occupied by humans and pets, control methods must be safe for people and the environment [60]. Applications of abamectin and mineral oil for *A. simplex* management pose minimal risk to humans and pets. Although abamectin is a conventional pesticide, it is typically applied at low rates in landscapes and degrades rapidly in the environment, minimizing exposure risks [2]. Furthermore, abamectin exhibits low toxicity to mammals, making it a suitable option for landscape applications [2].

Despite the promising performance of abamectin in controlling *A. simplex*, sole reliance on this conventional pesticide may raise several concerns. Resistance development has been documented in eriophyoid species such as *Phyllocoptruta oleivora* (Ashmead) [61], as well as in various mite and insect species, often linked to their high reproductive rates and short life cycle [62,63,64]. Abamectin exposure can be sublethal for some non-target arthropods, including predatory mites, which often results in reduced fecundity and shortened longevity [65,66]. Detrimental effects have been documented in *Amblyseius largoensis* (Muma), *Amblyseius swirskii* (Athias-Henriot), *Neoseiulus barkeri* (Athias-Henriot), *Neoseiulus californicus* (McGregor), *Neoseiulus cucumeris* (Oudemans), *Phytoseius intermedius* Evans and MacFarlane (Acari: Phytoseiidae), which are predator mite species, either commercially available or naturally occurring, widely implemented in biological control programs to mitigate eriophyoids [27,51,66,67,68,69,70]. Additionally, the combined effects of abamectin-induced resistance development and the compromised effectiveness of natural enemies may destabilize pest management efforts, ultimately leading to pest resurgence and secondary pest outbreaks [62,71]. Mineral oil overcomes these concerns as (i) eriophyid mites have not been reported to develop resistance to its multiple modes of action and (ii) it has fewer detrimental effects on non–target arthropods, including the parasitoids *Tamarixia radiata* (Waterston) (Hymenoptera: Eulophidae) and *Diaphorencyrtus aligarhensis* (Shafee, Alam & Agarwal) (Hymenoptera: Encyrtidae), the predator insect *Podisus nigrispinus* (Dallas) (Heteroptera: Pentatomidae), and the predator mite *Neoseiulus californicus* (McGregor) (Mesostigmata; Phytoseiidae) [49,72].

To address these limitations, abamectin and mineral oil can be used in rotation, as they have different modes of action, which may help delay resistance development in mite populations [51]. Mineral oil is a horticultural oil frequently used as an adjuvant to enhance the performance of conventional pesticides, such as abamectin. This synergistic combination improves spray penetration, increases plant surface coverage, and enhances translaminar movement through plant tissue, increasing the likelihood of the active ingredient penetrating galls (i.e., erinea) while also protecting it from photodegradation, thereby extending its residual activity [30,73]. For example, the combination of abamectin and mineral oil has been effective in controlling *A. litchii*, a species that also inhabits erinea [51]. Beyond its synergistic role, mineral oil represents a viable standalone alternative to abamectin, as it is a biorational pesticide rather than a synthetic chemical. Biorational pesticides contain naturally derived active ingredients and are considered environmentally sustainable due to their low mammalian toxicity, inability of arthropods to develop resistance to this mode of action, and minimal detrimental effects on predatory mites and other non-target organisms [49,74]. Mineral oil, however, would represent the only available tool capable of effectively suppressing and preventing *A*. *simplex* infestations, thus preserving the appearance of *R. simplex* [75]. Although other products such as garlic and thyme oils showed strong potential in laboratory assays, they were not further evaluated under greenhouse conditions due to concerns regarding their practicality in landscape use. This is because garlic oil has been associated with skin and eye irritation [76], raising safety concerns for its use in public or residential landscapes. Meanwhile, thyme oil was ineffective in controlling *A. simplex* populations within the erinea when applied curatively, and its use may still compromise the visual appearance of *R. simplex*, which is unacceptable in ornamental plants. Nevertheless, thyme oil has proven effective against eriophyoid mites, such as *Aculops cannibicola* Farkas [77], whose life cycle occurs on plant surfaces rather than within protective structures such as erinea. This increased exposure likely accounts for the higher susceptibility of *A. cannabicola* to thyme oil, in contrast to *A. simplex*, which remains concealed within the erinea and is consequently less affected by the treatment [78].

Although the results are promising, two methodological considerations should be acknowledged to properly contextualize the findings. First, the curative pesticide efficacy trials conducted under laboratory conditions were terminated at 72 h post-treatment. This decision was based on previous findings indicating that most *A. simplex* individuals emerge from the erinea within the first 24 to 72 h [13]. Considering that the typical life cycle of eriophyoid mites spans 5–10 days, the residual activity and long-term effects of treatments may have been underestimated. Second, pesticide efficacy in the laboratory was assessed from the number of mites emerging from the erinea rather than from direct observations on mortality, due to the difficulty of scoring mites inside the galls. This approach may have underestimated survival rates if viable individuals remained quiescent within the erinea. Nonetheless, these limitations are partially mitigated by our greenhouse findings, which corroborated the laboratory results by demonstrating reduced or prevented formation of new erinea following treatment applications, supporting the efficacy of the pesticides under more realistic conditions. To date, an IPM program for *A. simplex* in landscapes relies primarily on regular monitoring and chemical intervention. Monitoring efforts should focus on the youngest plant tissues, where *A. simplex* preferentially feeds and induces the formation of erinea. In the initial stage, the hyaline erinea are nearly transparent and may be overlooked or misidentified as plant trichomes. Due to this subtle appearance, infestations often remain undetected during early development. Detection typically occurs once the erinea turn white, indicating a more advanced stage of mite infestation [13]. As a tropical and subtropical species reported from Anguilla, Cuba, Brazil, the Dominican Republic, Florida, and Hawaii [10,11,13,79], *A. simplex* is not expected to overwinter, although this has not been formally documented. Therefore, monitoring throughout the year is essential to ensure early detection and effective management. Once an infestation is confirmed, applications of abamectin and mineral oil provide a reliable option for suppressing *A. simplex* populations and reducing erinea development. When considering control strategies, prophylactic applications in landscapes are often limited due to cost, environmental impact, and potential exposure risks for humans and pets [76]. Nevertheless, they may be justified in high-risk situations, especially when infested *R. simplex* plants are near uninfested ones. In such scenarios, simultaneous curative and prophylactic treatments can help suppress active infestations and limit further spread. Moreover, in nursery systems, where tolerance for visible damage on plants is extremely low [80], abamectin and mineral oil can also be applied preventively to protect uninfested stock and preserve marketability. Our findings represent a first step in developing a broader IPM program against *A. simplex*. Building on this foundation, a more comprehensive approach should integrate established cultural and biological control strategies commonly implemented to mitigate other eriophyoid mites. For example, cultural control could complement chemical control in mitigating *A. simplex*, as pruning infested plant tissue with erinea before pesticide applications has been shown to reduce infestations, in *A. litchii* and *Aceria reyesi* (Nuzzaci) [81,82]. Although pruning has been considered costly and inefficient if not followed by pesticide application [46,78], recent studies have demonstrated that it is an eco–friendly alternative for controlling eriophyoid infestations. Furthermore, several predatory mites have been shown to suppress erinose mites, such as *P. intermedius* on *A. litchii* [22,51]. However, abamectin has demonstrated detrimental effects when combined with predatory mites such as *P. intermedius* [51], indicating the need for careful integration of chemical and biological controls. Therefore, future studies should focus on prioritizing multitactical strategies that enhance the sustainability of IPM programs targeting *A. simplex* in ornamental landscapes. Findings from this research not only contribute to develop an IPM program against *A. simplex* but also provide a foundation for managing other eriophyoid species infesting ornamental landscapes.

## Figures and Tables

**Table 1:** List of commercial formulated biorational and conventional pesticides evaluated against *Acalitus simplex* under laboratory conditions.

Pesticide	Trade Name	Active Ingredient	Pesticide Group ^a^	Rate ^b^	Rate in 50 mL Solution	Site ^c^	EPA Registration Number ^d^
Biorational Pesticides	Bush Doctor Force of Nature	Garlic oil	Unclassified	1.53 L/ha	0.59 mL	G, L, N, S	FIFRA 25 (b) exempt
	NUKE EM^®^	Citric acid	Unclassified	0.096 L/ha	3.13 mL	G, L, N, S	FIFRA 25 (b) exempt
	Thyme^®^ Guard	Thyme oil	Unclassified	0.5%	0.25 mL	G, L, N, S	FIFRA 25 (b) exempt
	Suffoil–X^®^	Mineral Oil	Unclassified	2%	1 mL	G, L, N	48813–1–68539
	M–PEDE^®^	Potassium salts of fatty acids	Unclassified	0.03 L/ha	0.98 mL	G, I, L, N	10163–324
Conventional Acaricides/Insecticides	Avid 0.15EC	Abamectin	6	0.05 L/ha	0.016 mL	G, L, S	100–896
	Akari^®^ 5SC	Fenpyroximate	21A	0.29 L/ha	0.094 mL	N, I, L	71711–4
	Talstar^®^ P	Bifenthrin	3A	0.26 L/ha	0.08 mL	G, L, I	279–3206
	Forbid^®^ 4F	Spiromesifen	23	0.05 L/ha	0.016 mL	L	432–1279

^a^ Pesticide group is based on the IRAC mode of action classification; ^b^ Rate calculations are based on the maximum label-recommended amount of product to be applied to one hectare (ha); ^c^ Recommended application site for each product (S: shade house, G: greenhouse, N: nursery, L: landscape, I: interior); ^d^ All products are registered and commercially available for use in Florida against mites.

**Table 2:** Mean number of *Acalitus simplex* exposed to curative and prophylactic applications under laboratory conditions. In the curative application means refer to emerging mites from the erinea, while in the prophylactic means refer to dead mites.

Spray Application	Pesticide	Mean ± SE	95% Cl Lower	95% Cl Upper
Curative Application	Garlic oil	8.33 ± 2.16	5.01	13.85
	Citric acid	19.33 ± 4.78	11.91	31.38
	Thyme oil	17.26 ± 4.31	10.58	28.14
	Mineral Oil	9.29 ± 2.39	5.61	15.40
	Potassium salts of fatty acids	22.67 ± 5.57	14.00	36.69
	Abamectin	1.10 ± 0.41	0.53	2.27
	Fenpyroximate	22.43 ± 5.51	13.86	36.29
	Bifenthrin	39.37 ± 9.43	24.62	62.94
	Spiromesifen	31.92 ± 7.69	19.91	51.18
	Water	33.75 ± 8.14	21.04	54.13
Prophylactic Application	Garlic oil	12.09 ± 0.92	10.41	14.04
	Citric acid	5.01 ± 0.46	4.18	5.99
	Thyme oil	6.51 ± 0.56	5.50	7.71
	Mineral Oil	20.96 ± 1.48	18.25	24.08
	Potassium salts of fatty acids	5.57 ± 0.50	4.67	6.64
	Abamectin	22.24 ± 1.56	19.39	25.52
	Fenpyroximate	5.10 ± 0.47	4.26	6.11
	Bifenthrin	5.14 ± 0.47	4.30	6.15
	Spiromesifen	4.32 ± 0.41	3.58	5.22
	Water	1.40 ± 0.22	1.04	1.90

## Data Availability

Data files are available in the figshare repository: https://doi.org/10.6084/m9.figshare.29799959.v1.
